# Inhibition of TLR4/MAPKs Pathway Contributes to the Protection of Salvianolic Acid A Against Lipotoxicity-Induced Myocardial Damage in Cardiomyocytes and Obese Mice

**DOI:** 10.3389/fphar.2021.627123

**Published:** 2021-03-08

**Authors:** Zhen Yang, Yanli Chen, Zhaoyuan Yan, Tian Tian Xu, Xiangyao Wu, Aiwen Pi, Qingsheng Liu, Hui Chai, Songtao Li, Xiaobing Dou

**Affiliations:** ^1^ College of Basic Medicine and Public Health, Zhejiang Chinese Medical University, Hangzhou, China; ^2^ College of Life Science, Zhejiang Chinese Medical University, Hangzhou, China; ^3^ Molecular Medicine Institute, Zhejiang Chinese Medical University, Hangzhou, China; ^4^ Hangzhou Hospital of Traditional Chinese Medicine, Guangxing Hospital Affiliated to Zhejiang University of Traditional Chinese Medicine, Hangzhou, China

**Keywords:** salvianolic acid A, lipotoxicity, myocardial injury, TLR4, MAPKs

## Abstract

The occurrence of lipotoxicity during obesity-associated cardiomyopathy is detrimental to health. Salvianolic acid A (SAA), a natural polyphenol extract of *Salvia miltiorrhiza Bunge* (Danshen in China), is known to be cardioprotective. However, its clinical benefits against obesity-associated cardiomyocyte injuries are unclear. This study aimed at evaluating the protective effects of SAA against lipotoxicity-induced myocardial injury and its underlying mechanisms in high fat diet (HFD)-fed mice and in palmitate-treated cardiomyocyte cells (H9c2). Our analysis of aspartate aminotransferase and creatine kinase isoenzyme-MB (CM-KB) levels revealed that SAA significantly reversed HFD-induced myocardium morphological changes and improved myocardial damage. Salvianolic acid A pretreatment ameliorated palmitic acid-induced myocardial cell death and was accompanied by mitochondrial membrane potential and intracellular reactive oxygen species improvement. Analysis of the underlying mechanisms showed that SAA reversed myocardial TLR4 induction in HFD-fed mice and H9c2 cells. Palmitic acid-induced cell death was significantly reversed by CLI-95, a specific TLR4 inhibitor. TLR4 activation by LPS significantly suppressed SAA-mediated lipotoxicity protection. Additionally, SAA inhibited lipotoxicity-mediated expression of TLR4 target genes, including MyD88 and *p*-JNK/MAPK in HFD-fed mice and H9c2 cells. However, SAA did not exert any effect on palmitic acid-induced SIRT1 suppression and *p*-AMPK induction. In conclusion, our data shows that SAA protects against lipotoxicity-induced myocardial damage through a TLR4/MAPKs mediated mechanism.

## Introduction

Obesity is a global epidemic that is characterized by excessive amounts of circulating free fatty acids (FFAs), resulting in a variety of metabolic disorders, including lipotoxic cardiomyopathy (Schrammel et al., 2013). Lipotoxicity occurs when FFAs form heterotopic deposits in non-adipose tissues, including the kidney, skeletal muscles, and liver (Sletten et al., 2018). Lipotoxicity-induced myocardial damage accelerates advanced heart disease development. Palmitic acid (PA), the most abundant saturated fatty acid in human diet, has been found to be significantly elevated in blood samples of both obese individuals and high-fat diet (HFD) -fed animals (van Rooijen and Mensink, 2020). Therefore, PA is widely used to induce lipotoxicity *in vitro*.

Although the mechanisms underlying obesity-associated cardiac dysfunction are not fully understood, several factors, including toll-like-receptor 4 (TLR4) have been implicated. TLR4 is a well-known activator of innate immune responses against pathogens and has recently been shown to mediate obesity-induced cardiac inflammation, glucose metabolic derangement and injury (Jackson et al., 2015; Chen et al., 2020; Liu et al., 2020). Palmitate is an endogenous TLR4 ligand that significantly upregulates TLR4 levels in hepatocytes (Dou et al., 2020). Palmitate also enhances TLR4 expression in cardiomyocytes (Wang et al., 2017). Studies on HFD-induced obesity have shown that myocardial damage is significantly alleviated by TLR4 depletion (Wang et al., 2017). MyD88 is an adapter protein that mediates TLR4 signal transduction and activation of mitogen-activated protein kinase (MAPK) signaling pathway. MyD88 or MAPKs (including JNK and ERK1/2) inhibition has been shown to improve lipotoxicity-induced cardiac injury in both obese mice and cultured cardiomyocytes (Liu et al., 2014; Wang et al., 2016). Additionally, adenosine monophosphate activated protein kinase (AMPK), a key cellular energy sensor, has been implicated in HFD-induced cardiac hypertrophy, palmitate-induced apoptosis, and cardiomyocytes lipid deposit (Gélinas et al., 2018; Yang et al., 2018; Zhang et al., 2019). AMPK activation inhibits myocardial hypertrophy by regulating energy metabolism through sirtuin 1 (SIRT1) (Ma et al., 2018).

*Salvia miltiorrhiza Bunge* (Danshen in China) is a Chinese medicinal herb that has traditionally been used for the treatment of cardiovascular diseases. Salvianolic acid A (SAA) is the main water-soluble component of Danshen. It has been established that SAA has a range of pharmacological effects, including anti-oxidant, anti-inflammatory, anti-fibrotic, and anti-carcinogenic activities (Yang et al., 2012; Zheng et al., 2015; Wang et al., 2019a). Animal models have shown that SAA is cardioprotective, including during ischemic-reperfusion, cardiomyocyte infarction, and arsenic trioxide-induced injury (Ho and Hong, 2011; Feng et al., 2017; Wang et al., 2019b). However, its clinical benefits against lipotoxicity-induced myocardial damage have not been established.

In this study, we investigated the clinical benefits of SAA against lipotoxicity-induced cardiac injuries in both HFD-induced obese mice and palmitate-exposed cardiomyocytes. Our findings highlight the potential therapeutic values of SAA in the prevention and treatment of obesity-associated myocardial damage.

## Materials and Methods

### Animals Studies

Animal care and experiments complied with National Institute of Animal Health guidelines on animal research. Ethical approval was granted by the Animal Ethics Committee at Zhejiang Chinese Medical University. Eight-week old male C57BL/6J mice were housed at 22 ± 2°C and 12-h light/dark cycle. Animals were divided into the following groups (6 mice each); normal diet group (Control), HFD group (HFD), normal diet with 40 mg/kg BW SAA group (SAA), and HFD with 40 mg/kg BW SAA (SAA + HFD) group. The Control group was fed AIN-93G diet. HFD mice were fed 60% fat diet to induce obesity (60% fat, D12492, Research Diets, New Brunswick, NJ), and water enriched with high fructose and sucrose. 42 g/L of carbohydrates was added to drinking water at a ratio of 45% sucrose and 55% fructose (St. Louis, MO) by weight. SAA was dissolved in sterilized physiologic saline to a stock concentration of 20 mg/ml. 100 μL aliquots of the SAA solution or sterilized physiologic saline were intraperitoneally administered to mice every other day for 12 weeks. Body weight was recorded weekly. At the end of the experiment, mice were fasted overnight, anesthetized using sodium pentobarbital (50 mg/kg BW), and sacrificed to obtain plasma and hearts for analysis. The obese mice model was successfully established by the evaluating of body weight and plasma lipids (Supplementary Figure S1).

### Measurement of Plasma Lipids, AST, CK-MB Level

Mice were sacrificed under anesthesia, blood was obtained and plasma was harvested by centrifugation for 5 min at 3,000 rpm. Plasma AST, CK-MB, triglyceride (TG), free fatty acids (FFA), total cholesterol, high-density lipoprotein-cholesterol (HDL-C), low-density lipoprotein-cholesterol (LDL-C) levels were then measured using commercial kits (Nanjing Jiancheng BioTech. Co. Ltd., Nanjing, China) according to the manufacturers’ instructions.

### Immunohistochemistry

Hearts were fixed in 4% PFA and embedded in paraffin. Sectioned tissues were then stained using H&E and their myofibers analyzed histologically. H&E sections were examined and imaged under brightfield illumination on a Leica microscope (Carl Zeiss, Gottingen, Germany).

### Reagents

Lipopolysaccharides (LPS) was purchased from Selleck Chemicals (Huston, TX). PA, stearic acid (SA), oleic acid (OA), and SAA were purchased from Sigma-Aldrich (St. Louis, MO). CLI-95 was purchased from Invitrogen (Carlsbad, CA). SA-BSA and PA-BSA were processed as previously described (Dou et al., 2020). Briefly, SA or PA were solubilized in 100% ethanol and saponified with sodium hydroxide. The sodium salt was desiccated and resuspended in PBS. It was then heated at 80°C to achieve complete dissolution. We then added an isovolumetric 20% (w/v) BSA and stirred the mixture at 50°C for 4 h to allow SA or PA to bind BSA. The SA-BSA or PA-BSA complex (3 mmol/L fatty acid:1.5 mmol/L BSA, molar ratio, 2:1) was sterilized by filtration, and aliquoted for use. Oleate was complexed with 10% BSA to produce a stock solution, which was diluted in culture medium prior to use. The control and vehicle groups were treated with equal amounts of solvents (BSA or DMSO).

### Cell Culture

H9c2 cells were obtained from Shanghai Institute Cell Bank (Shanghai, China). Cells were cultured in DMEM medium (Gibco, Eggenstein, Germany) containing 5.5 mmol/L d-glucose and supplemented with 10% FBS (Biological Industries, ISR) as well as 100 U/mL/100 mg/ml pen/strep, at 37°C, in a humidified incubator, O_2_/CO_2_ (19:1) atmosphere.

### Cell Death Assay

2×10^5^ cells/well were seeded onto a 24-well plate and cultured in normal media or media containing indicated concentrations of palmitate in the presence or absence of SAA (5, 10, 20, 40 μM). After 12 h, 450 μM MTT solution was added into each well and incubated for 4 h. The supernatant was removed and 600 μL DMSO added to dissolve the resulting formazan. Absorbance was then read at 470 nm using FLUOstar Omega (BMG Labtech). For the LDH test, the culture medium was collected and LDH assayed using an LDH assay kit (Thermo Scientific Inc., VA) according to the manufacturers’ instructions. Nuclear morphological changes were examined by staining with 5 mg/L Hoechst 33342 for 10 min at 37°C before being imaged under a fluorescent microscope (Leica).

### Reactive Oxygen Species (ROS)

ROS levels were evaluated as described before (Li et al., 2014). After treatments, cells were washed and placed in serum free medium. 2, 7-dichlorodi-hydrofluorescein diacetate (DCFH-DA) was added to each well at a final concentration of 10 μM and cells cultured at 37°C for 20 min. They were then washed thrice using ice-cold PBS. Fluorescence intensity was determined using an inverted fluorescent microscope (Leica, Wetzlar, Germany). The mean fluorescence intensity (MFI) of five random fields was determined using ImageJ.

### Mitochondrial Membrane Potential (MMP) Assay

MMP was evaluated using Rhodamine123 (Rh123) at a final concentration of 100 μg/ml for 45 min. Fluorescence was measured using a fluorescent microscope (Leica, Wetzlar, Germany). The mean fluorescence intensity (MFI) of five random fields was determined using ImageJ.

### Western Blotting

Western blotting was done as previously described (Li et al., 2014) using the following antibodies: anti-β-actin, anti-cleaved-caspase3, anti-Bcl-2, anti-Bax, anti-MyD88, anti-JNK, anti-phospho-JNK, anti-ERK1/2, anti-phospho-ERK1/2, and anti-GAPDH from Cell Signaling Technology; Anti-TLR4, anti-SIRT1, anti-AMPK, anti-phospho-AMPK, from Santa Cruz Biotechnology. *β*-actin and GAPDH were used as loading controls. Protein levels were detected using HRP-conjugated secondary antibodies, and signal developed using ECL.

### Statistical Analysis

Data are presented as mean ± SD for ≥3 independent biological experiments with three replicates per experiment. Statistical analysis was done using SPSS. Student’s t-test or one-way ANOVA followed by Tukey test were used for pairwise comparisons between groups. *p* < 0.05 was set as the threshold for statistical significance.

## Results

### SAA Protects Against Lipotoxicity-Induced Myocardial Cell Injury

**FIGURE 1:**
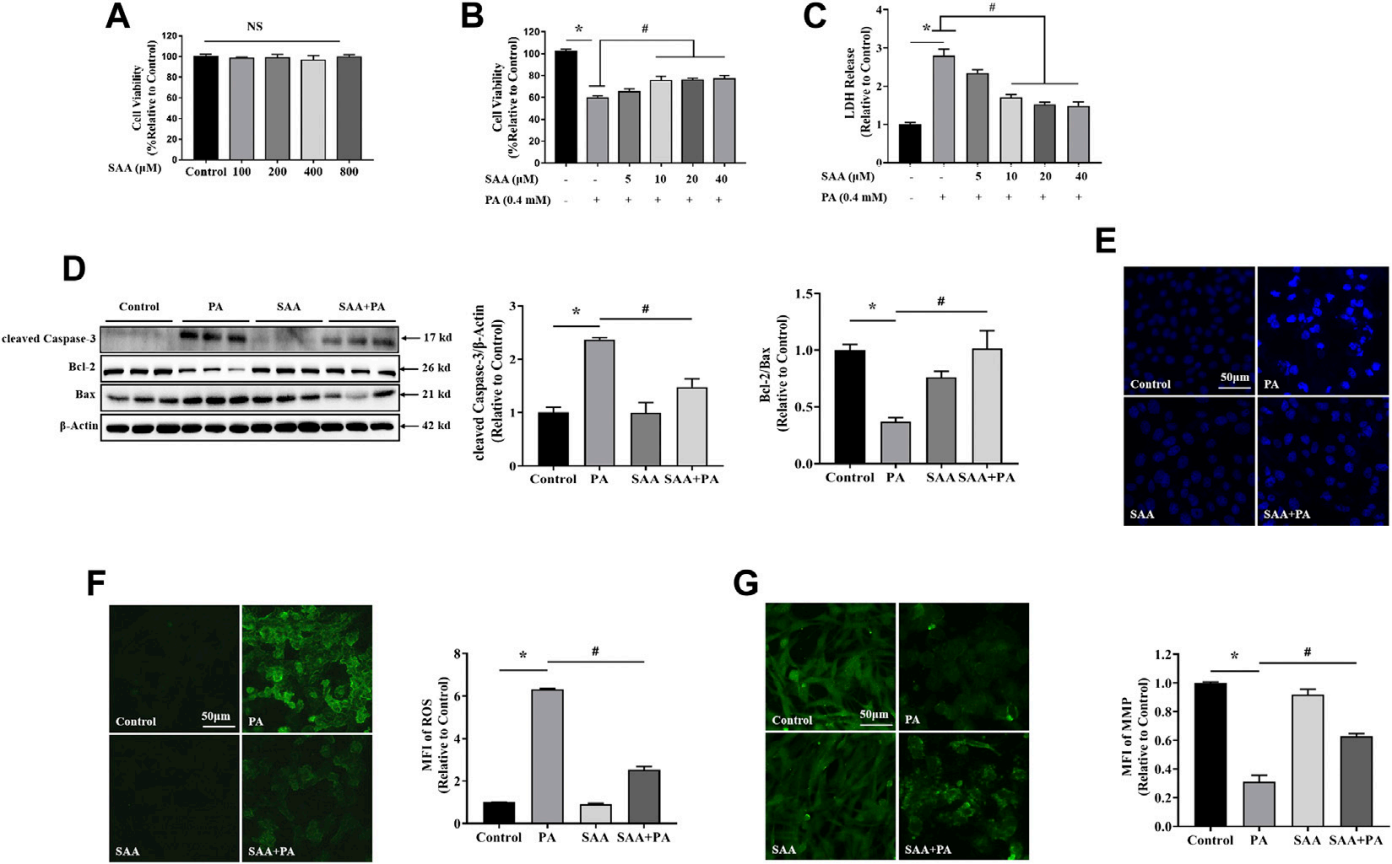
SAA reverses PA-induced lipotoxicity in H9c2 cardiomyocytes **(A)** Cardiomyocytes were seeded onto 24-well plates and grown to 80–90% confluence before treatment with the indicated SAA concentrations for 24 h. Cell viability was determined using MTT. **(B)**, **(C)** Cardiomyocytes were pretreated with indicated SAA concentrations for 1 h and incubated with 0.4 mM palmitic acid (PA) for 12 h and cell viability and LDH release measured. **(D)** Western blot analysis of cleaved Caspase-3, Bcl-2, and Bax. Values are presented as mean ± SD for three or more independent tests. **(E)** Hoechst staining for nuclei. Cell death was detected by morphologic examination after Hoechst staining by fluorescence microscopy (mag = 200X). **(F)** ROS levels were measured using DCFH-DA. **(G)** MFI analysis of mitochondrial membrane potential using Rh123. Values with different superscripts are significantly different at *p* < 0.05. * shows comparison with normal control group while # shows comparison with PA-only treatment group.

First, we tested the cytotoxic effects of various FFAs on myocardial cells with or without SAA. Analysis of LDH levels in the culture media revealed that long-chain saturated fatty acids (LSFAs), including PA and SA, exhibited significant dose-dependent lipotoxicity (Supplementary Figures S2A–D). OA, an important representative of long-chain monounsaturated fatty acids, did not exert lipotoxic effects in myocardial cells (Supplementary Figures S2E,F). Based on these results, we chose 0.4 mM PA to stimulate myocardial cells. To establish appropriate SAA concentrations for the experiments, we evaluated SAA cytotoxicity in cells without fatty acid treatment, and found that tolerance was up to 800 μM SAA (Figure 1A). Cell viability and LDH assays indicated that SAA significantly suppressed PA-induced cell death (Figures 1B,C). *In vitro* assays were done using SAA at a maximum concentration of 40 μM. Pretreating cells with SAA significantly reversed PA-induced cytotoxicity in a dose-dependent manner (Figures 1B,C). Then, 10 μM of SAA were chosen for the subsequent experiments. It was found that 10 μM SAA inhibited PA-mediated upregulation of cleaved Caspase-3, Bcl-2, Bax, and Bcl2/Bax ratio, indicating suppressed apoptosis (Figure 1D). Additionally, PA-stimulated chromatin damage was reversed by SAA pre-treatment (Figure 1E). Reduced circulating FFA levels enhanced oxidative stress during cardiomyopathy. This prompted us to assess SAA’s capacity to scavenge PA-induced ROS in cardiac cells. The DCFH-DA assay showed that 0.4 mM PA significantly increased ROS levels relative to control cells (Figure 1F). However, SAA was shown to significantly suppress ROS production. MFI values showed that SAA significantly reduced PA-induced ROS production. Pretreatment with SAA was also shown to significantly reverse PA-induced reduction of mitochondrial membrane potential (MMP) (Figure 1G). Together, these data demonstrate the protective effects of SAA against mitochondrial damage, apoptosis, and oxidative stress-mediated injury.

### The Protective Role of SAA Is Independent of SIRT1 and AMPK Pathways

**FIGURE 2:**
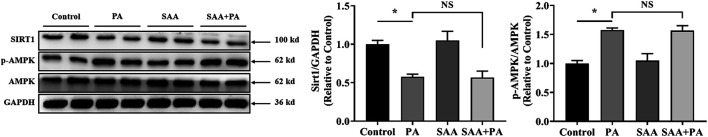
SIRT1/AMPK pathway is not involved in SAA protection from lipotoxicity. Cardiomyocytes were incubated with 0.4 mM palmitic acid (PA) for 12 h. 10 μM SAA was added 1 h before PA treatment. SIRT1, AMPK, and *p*-AMPK, protein levels were determined by western blot. Data were quantified by densitometric analysis as fold changes. Values are presented as mean ± SD for ≥3 independent tests. Bars with different characters are significantly different, *p* < 0.05.

Next, we determined whether SIRT1 mediates the beneficial effects of SAA against PA-induced cardiac lipotoxicity and found that SIRT1 levels were significantly reduced in PA-treated cells, while SAA treatment did not reverse SIRT1 suppression by PA (Figure 2). Analysis of potential AMPK involvement showed that pretreatment with SAA did not effectively improve PA-induced alteration of AMPK (Figure 2), implying that SIRT1 and AMPK did not mediate SAA effects in myocardial cells.

### Activation of TLR4/AMPK Signaling Pathway Contributes to SAA Lipo-Protective Effects in Cardiac Cells

**FIGURE 3:**
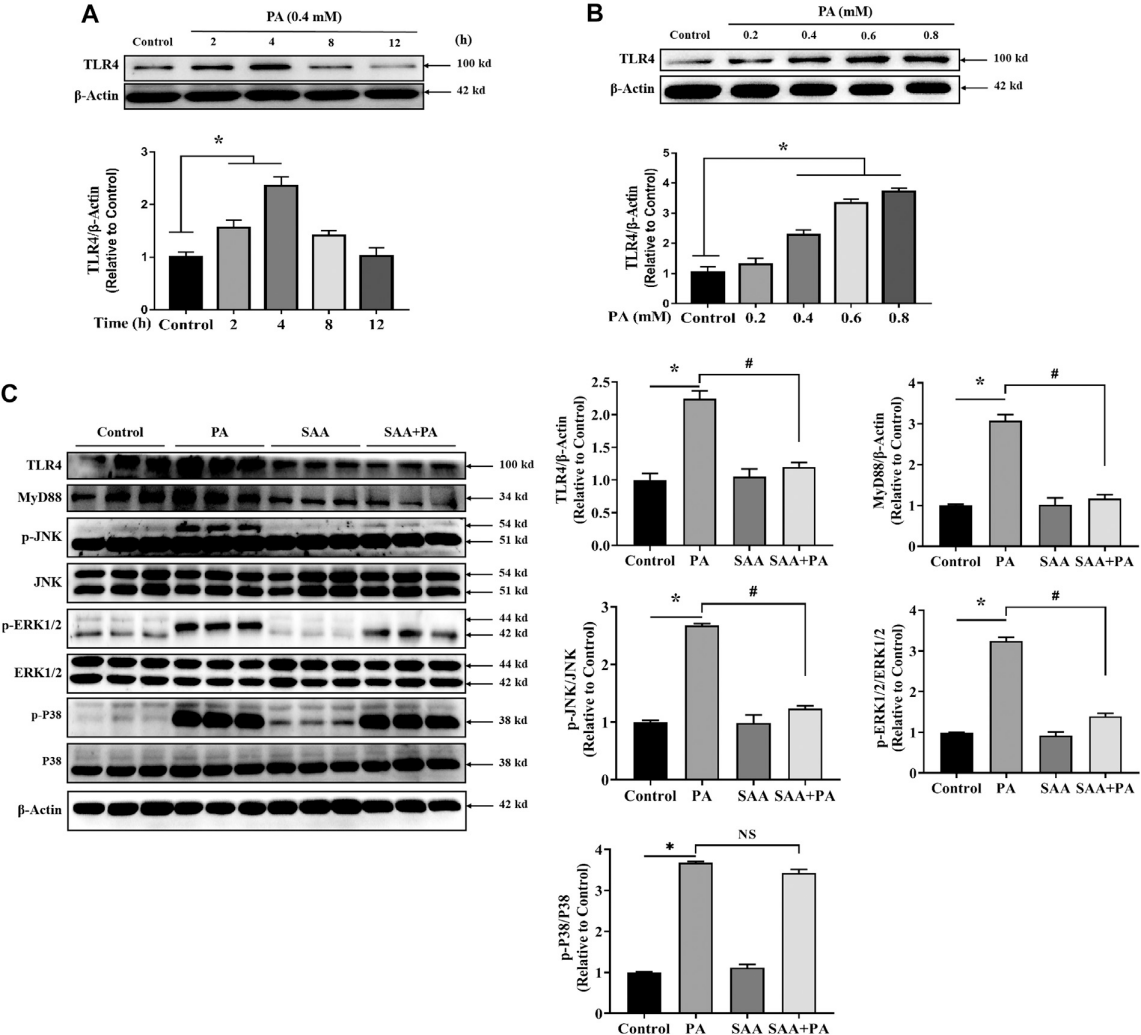
SAA attenuates palmitate-induced TLR4 activation. Cardiomyocytes were seeded onto 24-well plates and grown to 80% confluence before treatment with 0.4 mM PA for 2, 4, 8 and 12 h **(A)** or 0.2, 0.4, 0.6, or 0.8 mM PA for 4 h **(B)** For the detection of anti-lipotoxicity role of SAA, H9c2 cells were incubated with 0.4 mM palmitic acid (PA) for 4 h. 10 μM SAA was added 1 h before free fatty acids treatment. Representative western blot data on TLR4 levels. Data are shown as mean ± SD. Total protein was extracted from myocardial cells. TLR4, MyD88, *p*-JNK, and *p*-ERK1/2 levels were determined by western blot. Values with different superscripts are significantly different at *p* < 0.05. * indicates comparison with normal control group while # indicates comparison with PA-only treatment group.

TLR4 inhibition is thought to be an SAA target during liver injury. Therefore, we determined if TLR4 signaling modulated the protective effects of SAA against lipotoxicity. Analysis of TLR4 expression levels in PA-treated cardiac cells showed that treatment with PA at 0.4 mM for 2–4 h significantly increased TLR4 levels. At 8–12 h, the expression levels of TLR4 were not found to be significantly different from the untreated cardiac cells (Figure 3A). Then, we determined the expression level of TLR4 after the treatment with indicated concentrations (0.2, 0.4, 0.6, 0.8 mM) of *p*A. It was found that TLR4 were significantly up-regulated by PA treatment in a dose-dependent manner (Figure 3B). We then tested 0.4 mM concentration of PA on H9c2 cells for 4 h with or without SAA. MAPKs mediate SAA protection of liver injury. Analysis of whether MAPKs signaling mediate SAA protection against PA-induced lipotoxicity showed that SAA pretreatment significantly reversed PA-induced upregulation of MyD88, *p*-JNK and *p*-ERK1/2 levels. This implies that SAA lipo-protective effects are, partly, mediated by TLR4/MyD88/MAPKs signaling (Figure 3C).

### TLR4 Upregulation Mediates PA-Induced Lipotoxicity in Cardiac Cells

**FIGURE 4:**
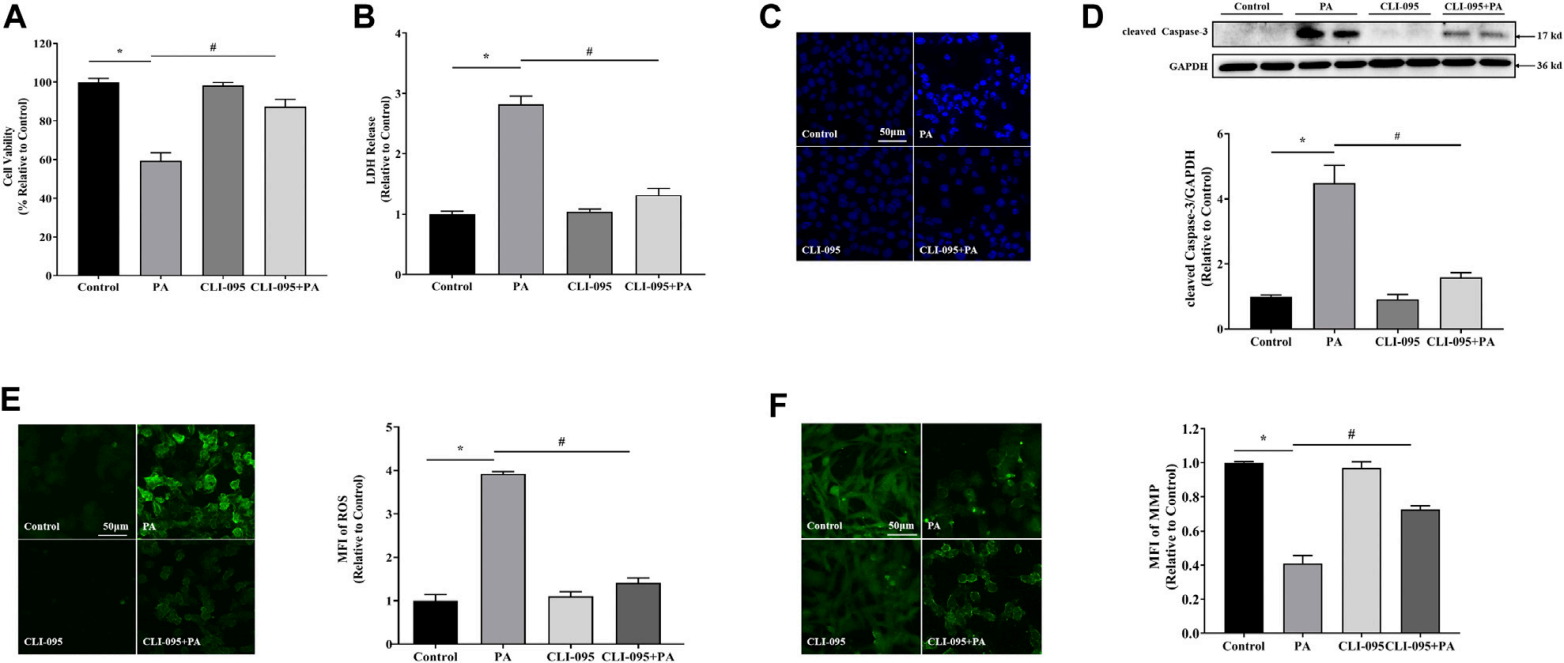
TLR4 was involved in lipotoxicity-induced injury in cardiomyocytes. Cardiomyocyte cells were treated with PA at 0.4 mM for 4 h with or without 1 h CLI-095 preincubation (1 μM). PA-induced cytotoxicity in CLI-095-treated cardiomyocytes was determined using the MTT assay **(A)** and analysis of LDH release into culture medium **(B)**. **(C)** Nuclear staining with Hoechst. **(D)** Representative western blot with densitometric analysis of cleaved Caspase-3 levels in cells. **(E)** ROS levels were measured using DCFH-DA. **(F)** MFI analysis of mitochondrial membrane potential using Rh123. Values are presented as mean ± SD for ≥3 independent tests. * indicates comparison with normal controls while # shows comparison with PA-only treatment. Bars with different characters are significantly different, *p* < 0.05.

To confirm whether TLR4 regulates lipotoxicity in cardiomyocytes, we inhibited TLR4 using CLI-095 in H9c2 cells. TLR4 inhibition significantly suppressed cytotoxicity and LDH release relative to palmitate-treated cells (Figures 4A,B), supporting the observation that TLR4 facilitates PA-induced lipotoxicity in cardiomyocytes. Moreover, inhibiting TLR4 also suppressed PA-induced elevation of cleaved Caspase-3 and ROS, mitochondrial membrane potential repression, and apoptosis in cardiac cells (Figures 4C–F), implying that TLR4 is involved in PA-induced lipotoxicity in cardiac cells.

### TLR4/MAPKs Is Involved in SAA Lipo-Protective Effects in Cardiomyocytes

**FIGURE 5:**
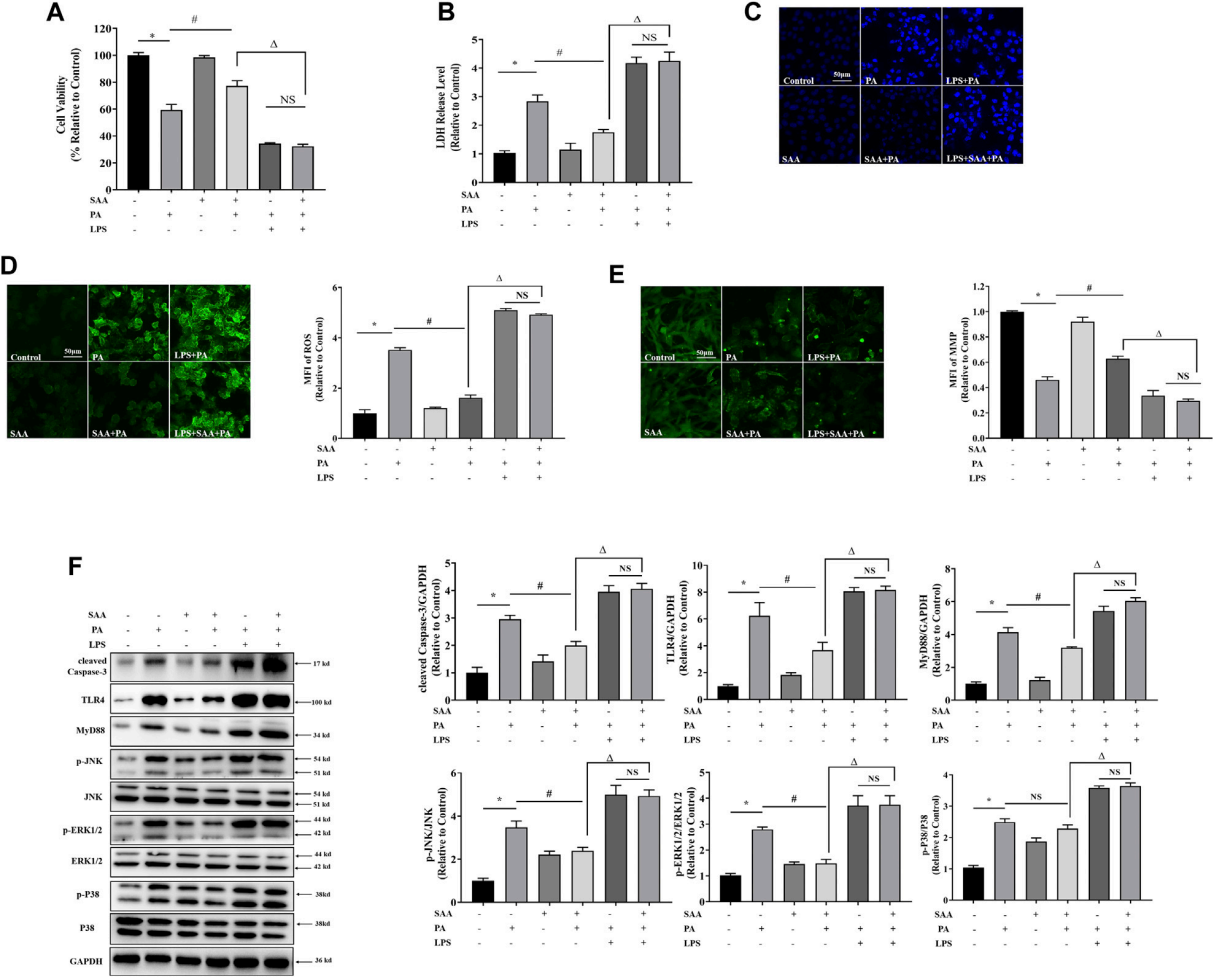
TLR4 activation contributes to SAA protection from lipotoxicity-induced injury in cardiomyocytes. Cardiomyocyte cells were treated with PA at 0.4 mM for 4 h. TLR4 was activated with 100 ng/ml LPS for 2 h before PA treatment. 10 μM SAA was added 1 h before PA treatment. **(A)** Cell viability was tested using MTT. **(B)** LDH release measurement. **(C)** Nuclear staining with Hoechst. Cell death was detected by assessing nuclear morphology by fluorescence microscopy at a ×200 magnification **(D)** ROS levels were measured by DCFH-DA. **(E)** Mitochondrial membrane potential (MMP) was examined by fluorescence microscopy. **(F)** Cleaved Caspase-3, TLR4, MyD88, *p*-JNK, and *p*-ERK1/2 levels were determined by western blot. Values are presented as mean ± SD for ≥3 independent tests. Bars with different characters are significantly different, *p* < 0.05.

To determine the role of TLR4/MAPKs in SAA’s lipo-protective effects in cardiomyocytes, we activated TLR4 using LPS and found that it significantly suppressed the protective properties of SAA against lipotoxicity, including cell viability, LDH release, MMP, ROS formation, apoptosis, and cleaved Caspase-3 levels (Figures 5A–F). Moreover, TLR4 upregulation mitigated the lipo-protective effects of SAA through PA-induced targets of MyD88 and MAPKs, including *p*-JNK and *p*-ERK1/2 (Figure 5F). These findings confirmed that TLR4 signaling is involved in the exertion of the lipo-protective effects of SAA in cardiomyocytes.

### TLR4/MAPKs Mediate SAA Lipo-Protective Effects in Cardiomyocytes of HFD-Fed Mice

**FIGURE 6:**
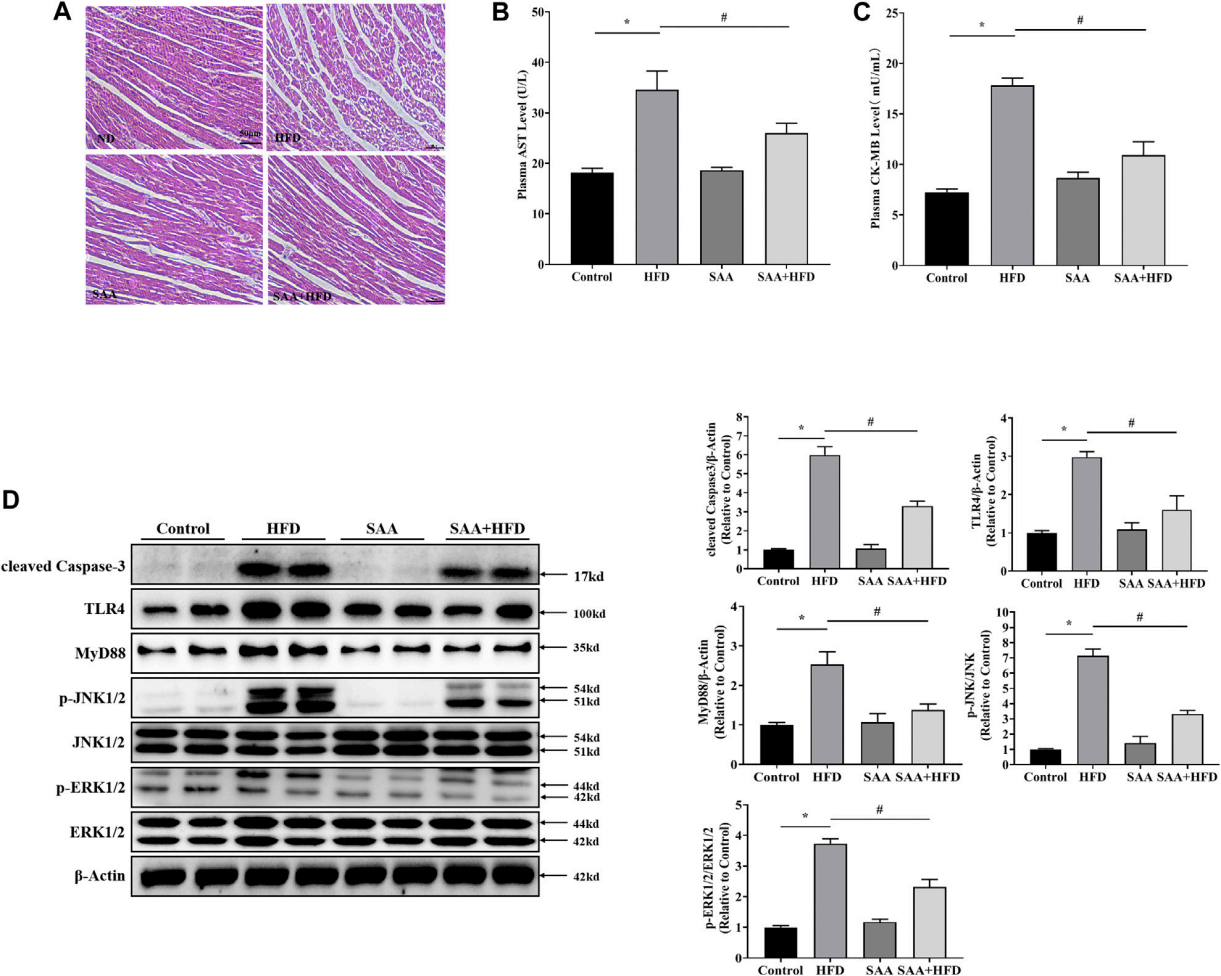
TLR4/MAPKs pathway is involved in SAA protection of cardiomyocytes from lipotoxicity-induced injury in high-fat diet (HFD)-fed mice. Animals were divided into the following groups (6 mice each); normal diet group (Control), HFD group (HFD), normal diet with 40 mg/kg BW SAA group (SAA), and HFD with 40 mg/kg BW SAA (SAA + HFD) group. The Control group was fed AIN-93G diet. HFD mice were fed 60% fat diet to induce obesity (60% fat, D12492, Research Diets, New Brunswick, NJ) and water enriched with high fructose and sucrose. SAA was dissolved in sterilized physiologic saline with a stock concentration of 20 mg/mL. A total volume of 100 μL SAA diluted solution or sterilized physiologic saline was intraperitoneally administered every other day for 12 weeks. **(A)** H/E staining of cardiomyocytes - scale bars = 50 μm. **(B)** Plasma aspartate aminotransferase (AST) level **(C)** plasma creatine kinase, MB (CK-MB) level. **(D)** Cleaved Caspase-3, TLR4, MyD88, JNK, *p*-JNK, ERK1/2 and *p*-ERK1/2 levels in mouse heart tissues were determined by western blot. Values with different superscripts are significantly different at *p* < 0.05. * indicates comparison with normal controls while # shows comparison with HFD-fed group.

To determine the protective effects of SAA in cardiomyocytes of HFD-fed mice, we examined the pathological alterations of cardiomyocytes in various groups using H&E staining. There were structural abnormalities in the cardiomyocytes of HFD-fed mice, including altered cellular structures and broken fibers, which were significantly alleviated by SAA treatment (Figure 6A). The plasma levels of AST and CK-MB are indicators of cardiomyocyte injury. We found that SAA significantly reduced HFD-driven increase in plasma AST and plasma CK-MB levels (Figures 6B,C). Furthermore, SAA significantly suppressed HFD-induced increase in cleaved Caspase-3 in mouse myocardial tissues (Figure 6D), implying that SAA prevented against cardiomyocyte lipotoxicity in HFD fed mice.

We then examined the role of TLR4/MAPKs in SAA anti-lipotoxic effects *in vivo* and found that SAA significantly suppressed HFD-induced elevation of TLR4, MyD88, *p*-JNK and *p*-ERK1/2, consistent with our *in vitro* data (Figure 6D). These findings suggest that TLR4/MAPKs inhibition contributes to SAA’s protection of cardiomyocytes from lipotoxicity.

## Discussion

We have shown that SAA, a phytochemical found in Danshen, protects against lipotoxicity in HFD-fed mice and cultured cardiomyocytes. Its benefits are associated with its effects on TLR4/MAPKs signaling. SAA is a bioactive component of *Salvia miltiorrhiza Bunge*. In traditional Chinese medicine, Danshen is used to enhance blood flow, resolve blood stasis and for the prevention and treatment of cardiovascular diseases. However, the cardioprotective benefits of SAA are not well understood.

Lipotoxicity refers to cellular damage and death caused by free fatty acids elevation and aberrant lipid metabolism. Lipotoxicity by saturated fatty acids (SFAs) promotes myocardial lipotoxic injury. Sustained SFA elevation leads to myocardial lipotoxic injury, resulting in cardiac dysfunction (Wende and Abel, 2010). As documented in literature, PA and SA, but not OA, cause significant myocardial cell injury by suppressing cell viability and enhancing LDH activity in a dose-dependent manner. Moreover, PA elevated Bcl2: Bax ratio, as well as Caspase-3 levels, implying that it mediates cardiomyocyte apoptosis. These data established that SFAs induce myocardial lipotoxic injury *in vitro*. LSFA induction of myocardial injury, mitochondrial dysfunction, and subsequent mitochondrial apoptosis mediate cardiomyopathic pathogenesis (Murphy et al., 2016). We found that cardiomyocytes are susceptible to PA stimulation, causing mitochondrial damage, increased ROS, decreased MMP, and reduced Bcl2/Bax ratio. To our knowledge, a limited number of studies have evaluated the protective effects of SAA against SFA-induced myocardial lipotoxicity. Analysis of the effect of SAA on PA-induced myocardial lipotoxicity revealed that SAA exposure inhibited the PA-induced LDH release and cell viability in a dose-dependent manner by reversing the effect of lipotoxicity on MMP, ROS, and Bcl2/Bax ratio. These findings are in tandem with the role of SAA on PA-induced hepatic lipotoxic injury in NAFLD (Li et al., 2020).

Activation of SIRT1/AMPK signaling may prevent oxidative stress and inflammation during lipotoxicity-related hepatocyte injury (Xu et al., 2020). SAA alleviates insulin resistance and HFD-induced NAFLD through AMPK activation (Li et al., 2020). Moreover, SIRT1/AMPK attenuates palmitate-induced oxidative stress in cardiomyocytes. Multiple bioactive compounds, including curcumin (Zhang et al., 2019) and resveratrol (Feng et al., 2019) protect cardiomyocytes from apoptosis through the AMPK pathway. However, in this study, SAA did not reverse PA-induced activation of SIRT1 and AMPK phosphorylation in cardiomyocytes.

Another potential mechanism for SAA is through the TLR4 axis (Yang et al., 2019). In myocardial cells, TLR4 acts as a cellular injury sentinel and mediates inflammatory responses (Zhou et al., 2020). PA has been reported to be a TLR4 ligand during the induction of myocardial inflammatory responses where it activates TLR4 after HFD-induced injury (Wang et al., 2017). In line with the reports, we found TLR4 expression levels were upregulated in PA-stimulated cardiomyocytes injury, *in vitro*. In addition, TLR4 inhibition using CLI-095 significantly suppressed PA-induced cardiomyocytes mitochondrial dysfunction and apoptosis. This implies that TLR4 inhibition is a potential therapeutic target for lipotoxic heart disease. Interestingly, the cytoprotective effects of SAA in various cellular contexts are attributed to TLR4 inhibition (Zeng et al., 2020). Through modulating TLR4 signaling, SAA reduces oxidative stress, inflammation, and apoptosis in liver tissue during insulin resistance (Yang et al., 2019). Thus, we determined whether TLR4 signaling involved in the protection of SAA against lipotoxicity-induced myocardial damage. Accordingly, SAA significantly alleviated PA-activated TLR4 protein levels, concurrent with reduction of its downstream factor MyD88 to activate the MAPK family members.

In cardiomyopathy, MAPK signaling activation induces cardiomyocytes apoptosis and promotes inflammation injury (Liang et al., 2019). *In vitro*, MAPKs overactivation has been reported to be involved in LSFAs-induced apoptosis in various cell types, including cardiomyocytes, pharmacologically inhibiting MAPKs or silencing them significantly elevates H_2_O_2_ levels and LSFAs-induced cell death (Oh et al., 2014). Previously, we have shown that TLR4/MAPK activation contributes to PA-induced cell death in hepatocytes. Analysis of if TLR4/MAPK signaling is mechanistically involved in PA-induced cell death in cardiomyocytes and SAA′ s cardioprotective effects found that PA upregulated TLR4/MAPKs expression in H9c2 cells *in vitro*, SAA reversed the expression of TLR4, MAPK and its downstream signals *p*-JNK, ERK1/2.

To further delinate the role of TLR4 in SAA and PA-induced lipotoxicity in cardiomyocytes lipotoxicity, we further dissected potential underlying mechanisms. In our previous study (Dou et al., 2020), activating TLR4 using LPS markedly blocked the lipid-reducing effects of Salidrosid. Consistent with the results, the TLR4 activation by LPS prominently abolished the beneficial role of SAA on TLR4-regulated MAPKs inhibition and myocardial apoptosis, which indicated that TLR4-regulated MAPKs pathway was mechanistically involved in SAA protection against cardiomyocytes lipotoxicity. However, we still do not know how SAA regulates TRL4/MAPKs expression in cardiomyocytes. In addition, LPS is a very strong activator of TLR4 pathway, which induction markedly aggravated PA-induced adverse effects, which might mask the potential protective effect of SAA through other pathways. Therefore, the participation of other potential signaling pathways in SAA-prevented lipotoxicity could also not be excluded, although it seems like that LPS completely abolished the beneficial effects of SAA.

In summary, we show that SAA has anti-inflammatory and anti-mitochondrial dysfunction effects *in vitro* and *in vivo*. Thus, the clinical benefits of SAA are closely associated with its ability to reduce TLR4 and inhibit MAPKs. We provide important insights into the mechanisms by which SAA-mediates myocardial protection against lipotoxic injury, thereby highlighting SAA as a potential dietary supplement or therapeutic agent for preventing and treating lipotoxicity-induced heart diseases.

## Data Availability

The original contributions presented in the study are included in the article/Supplementary Material, further inquiries can be directed to the corresponding authors.

## References

[B1] Chen Y. Feng B. Yuan Y. Hu J. Zhao W. Jiang H. (2020). Aloe emodin reduces cardiac inflammation induced by a high-fat diet through the TLR4 signaling pathway. Mediat. Inflamm. 2020, 6318520. 10.1155/2020/6318520 PMC7025072 32089647

[B2] Dou X. Ding Q. Lai S. Jiang F. Song Q. Zhao X. (2020). Salidroside alleviates lipotoxicity-induced cell death through inhibition of TLR4/MAPKs pathway, and independently of AMPK and autophagy in AML-12 mouse hepatocytes. J. Funct. Foods 65. 10.1016/j.jff.2019.103691

[B3] Feng L. Ren J. Li Y. Yang G. Kang L. Zhang S. (2019). Resveratrol protects against isoproterenol induced myocardial infarction in rats through VEGF-B/AMPK/eNOS/NO signaling pathway. Free Radic. Res. 53 (1), 82–93. 10.1080/10715762.2018.1554901 30526144

[B4] Feng S. Nan A. Geng J. Huang J. Sun R. Chun G. (2017). Pharmacokinetic and metabolomic analyses of the neuroprotective effects of salvianolic acid A in a rat ischemic stroke model. Acta Pharmacol. Sin. 38 (11), 1435–1444. 10.1038/aps.2017.114 28836583 PMC5672069

[B5] Gélinas R. Mailleux F. Dontaine J. Bultot L. Demeulder B. Ginion A. (2018). Bertrand bouchard, christine des rosiers, benoit viollet, kei sakamoto, jean-luc balligand, jean-louis vanoverschelde, christophe beauloye, sandrine horman, luc BertrandAMPK activation counteracts cardiac hypertrophy by reducing O- GlcNAcylation. Nat. Commun. 9, 374. 10.1038/s41467-017-02795-4 29371602 PMC5785516

[B6] Ho J. H. C. Hong C. Y. (2011). Salvianolic acids: small compounds with multiple mechanisms for cardiovascular protection. J. Biomed. Sci. 18 (1), 30. 10.1186/1423-0127-18-30 21569331 PMC3113734

[B7] Jackson E. E. RendinaRuedy E. Smith B. J. Lacombe V. A. (2015). Loss of toll-like receptor 4 function partially protects against peripheral and cardiac glucose metabolic derangements during a long-term high-fat diet. PloS One 10 (11), e0142077. 10.1371/journal.pone.0142077 26539824 PMC4634760

[B8] Li S. Li J. Shen C. Zhang X. Sun S. Cho M. (2014). tert-Butylhydroquinone (tBHQ) protects hepatocytes against lipotoxicity via inducing autophagy independently of Nrf2 activation. Biochim. Biophys. Acta 1841 (1), 22–33. 10.1016/j.bbalip.2013.09.004 24055888 PMC3884638

[B9] Li S. Qian Q. Ying N. Lai J. Feng L. Zheng S. (2020). Activation of the AMPK-SIRT1 pathway contributes to protective effects of Salvianolic acid A against lipotoxicity in hepatocytes and NAFLD in mice. Front. Pharmacol. 30 (11), 560905. 10.3389/fphar.2020.560905 PMC7734334 33328983

[B10] Liang Y. Lp M. S. M. Mak J. C. W. (2019). (-)-Epigallocatechin-3-gallate suppresses cigarette smoke-induced inflammation in human cardiomyocytes via ROS-mediated MAPK and NF-κB pathways. Phytomedicine 58 (5), 152768. 10.1016/j.phymed.2018.11.028 31005721

[B11] Liu H. Jia W. Tang Y. Zhang W. Qi J. Yan J. (2020). Inhibition of MyD88 by LM8 attenuates obesity-induced cardiac injury. J. Cardiovasc. Pharmacol. 76 (1), 63–70. 10.1097/FJC.0000000000000846 32398475

[B12] Liu Z. Wang J. Qiu C. Guan G. Liu X. Li S. (2014). Matrine pretreatment improves cardiac function in rats with diabetic cardiomyopathy via suppressing ROS/TLR-4 signaling pathway. Acta Pharmacol. Sin. 36 (3), 323–333. 10.1038/aps.2014.127 PMC4349921 25619390

[B13] Ma Z. Kong C. Song P. Zhang X. Yuan Y. Tang Q. (2018). Geniposide protects against obesity-related cardiac injury through AMPKα- and sirt1-dependent mechanisms. Oxid. Med. Cell Longev. 2018, 6053727. 10.1155/2018/6053727 30533173 PMC6247476

[B15] Murphy E. Ardehali H. Balaban R. S. DiLisa F. Dorn G. W. Kitsis R. N. (2016). Mitochondrial function, biology, and role in disease: a scientific statement from the American heart association. Circ. Res. 118 (12), 1960–1991. 10.1161/RES.0000000000000104 27126807 PMC6398603

[B17] Oh C. C. Nguy M. Q. Schwenke D. C. Migrino R. Q. Thornburg K. Reaven P. (2014). P38αmitogen-activated kinase mediates cardiomyocyte apoptosis induced by palmitate. Biochem. Biophys. Res. Commun. 450 (1), 628–633. 10.1016/j.bbrc.2014.06.023 24931668

[B18] Schrammel A. Mussbacher M. Winkler S. Haemmerle G. Stessel H. Wölkart G. (2013). Cardiac oxidative stress in a mouse model of neutral lipid storage disease. Biochim. Biophys. Acta 1831 (11), 1600–1608. 10.1016/j.bbalip.2013.07.004 23867907 PMC3795454

[B19] Sletten A. C. Peterson L. R. Schaffer J. E. (2018). Manifestations and mechanisms of myocardial lipotoxicity in obesity. J. Intern. Med. 284 (5), 478–491. 10.1111/joim.12728 29331057 PMC6045461

[B20] van Rooijen M. A. Mensink R. P. (2020). Palmitic acid versus stearic acid: effects of interesterification and intakes on cardiometabolic risk markers-A systematic review. Nutrients 12 (3), 615. 10.3390/nu12030615 PMC7146500 32111040

[B21] Wang R. Song F. Li S. Wu B. Gu Y. Yuan Y. (2019a). Salvianolic acid A attenuates CCl4-induced liver fibrosis by regulating the PI3K/AKT/mTOR, Bcl-2/Bax and caspase-3/cleaved caspase-3 signaling pathways. Drug Des. Dev. Ther. 13, 1889–1900. 10.2147/DDDT.S194787 PMC6549412 31213776

[B22] Wang R. Zhang J. Wang S. Wang M. Ye T. Du Y. (2019b). The cardiotoxicity induced by arsenic trioxide is alleviated by salvianolic acid A via maintaining calcium homeostasis and inhibiting endoplasmic reticulum stress. Molecules 24 (3), 543. 10.3390/molecules24030543 PMC6384753 30717322

[B23] Wang S. Ding L. Ji H. Xu Z. Liu Q. Zheng Y. (2016). The role of p38 MAPK in the development of diabetic cardiomyopathy. Int. J. Mol. Sci. 17 (7), 1037. 10.3390/ijms17071037 PMC4964413 27376265

[B24] Wang Y. Qian Y. Fang Q. Zhong P. Li W. Wang L. (2017). Saturated palmitic acid induces myocardial inflammatory injuries through direct binding to TLR4 accessory protein MD2. Nat. Commun. 8, 13997. 10.1038/ncomms13997 28045026 PMC5216130

[B25] Wende A. R. Abel E. D. (2010). Lipotoxicity in the heart. Biochim. Biophys. Acta 1801 (3), 311–319. 10.1016/j.bbalip.2009.09.023 19818871 PMC2823976

[B26] Xu H. Chen G. Ma Y. Zhang H. Zhou Y. Liu G. (2020). Hepatic proteomic changes and Sirt1/AMPK signaling activation by oxymatrine treatment in rats with non-alcoholic steatosis. Front. Pharmacol. 216 (11), 216. 10.3389/fphar.2020.00216 PMC7076077 32210812

[B27] Yang H. Feng A. Lin S. Yu L. Lin X. Yan X. (2018). Fibroblast growth factor-21 prevents diabetic cardiomyopathy via AMPK-mediated antioxidation and lipid-lowering effects in the heart. Cell Death Dis. 9 (2), 227. 10.1038/s41419-018-0307-5 29445083 PMC5833682

[B28] Yang L. Jiang L. Jiang D. Liu B. Jin S. (2019). The protective effects of salvianolic acid A against hepatic ischemia-reperfusion injury via inhibiting expression of toll-like receptor 4 in rats. Arch. Med. Sci. 15 (6), 1599–1607. 10.5114/aoms.2019.87412 31749890 PMC6855152

[B29] Yang L. Li D. Zhang Y. Zhu M. Chen D. Xu T. (2012). Salvianolic acid A inhibits angiotensin II-induced proliferation of human umbilical vein endothelial cells by attenuating the production of ROS. Acta Pharmacol. Sin. 33 (1), 41–48. 10.1038/aps.2011.133 22101169 PMC4010265

[B30] Zeng X. Chen X. Qin H. Han Y. Chen X. Han Z. (2020). Preventive effects of a natural anti-inflammatory agent Salvianolic acid A on acute kidney injury in mice. Food Chem. Toxicol. 135 (1), 110901. 10.1016/j.fct.2019.110901 31654708

[B31] Zhang J. Wang Y. Bao C. Liu T. Li S. Huang J. (2019). Curcumin-loaded PEG-PDLLA nanoparticles for attenuating palmitate-induced oxidative stress and cardiomyocyte apoptosis through AMPK pathway. Int. J. Mol. Med. 44 (2), 672–682. 10.3892/ijmm.2019.4228 31173176 PMC6605976

[B32] Zheng X. Chen S. Yang Q. Cai J. Zhang W. You H. (2015). Salvianolic acid A reverses the paclitaxel resistance and inhibits the migration and invasion abilities of human breast cancer cells by inactivating transgelin. Canc. Biol. Ther. 16 (9), 1407–1414. 10.1080/15384047.2015.1070990 PMC4622441 26176734

[B33] Zhou R. Gao J. Xiang C. Liu Z. Zhang Y. Zhang J. (2020). Salvianolic acid A attenuated myocardial infarction-induced apoptosis and inflammation by activating Trx. Naunyn-Schmiedeberg’s Arch. Pharmacol. 393 (6), 991–1002. 10.1007/s00210-019-01766-4 31811327

