# Supplementary material for: Inhibition of TLR4/MAPKs Pathway Contributes to the Protection of Salvianolic Acid A Against Lipotoxicity-Induced Myocardial Damage in Cardiomyocytes and Obese Mice
Source: Front Pharmacol. 2021 Mar 8;12:627123. doi: 10.3389/fphar.2021.627123 (PMC7982403; doi:10.3389/fphar.2021.627123)
Supplement: Supplementary file 1 [file datasheet1.doc]

**Supplementary Figure.1.** HFD-induced increase in body weight and plasma lipids content in C57BL/6J mice. (A) Dynamic alteration of body weight. (B) Morphological photographs of the mice. (C) Plasma triglycerides (TG) level. (D) Plasma free fatty acids (FFA) level. (E) Plasma total-cholesterol level. (F) Plasma high-density lipoprotein-cholesterol (HDL-C) level. (G) plasma low-density lipoprotein-cholesterol (LDL-C) level. All values are denoted as means ± SD. The values with different superscripts are significantly different at *P*＜0.05. * Comparisons with normal diet (ND) group. All groups contain 6 animals (n=6).

**
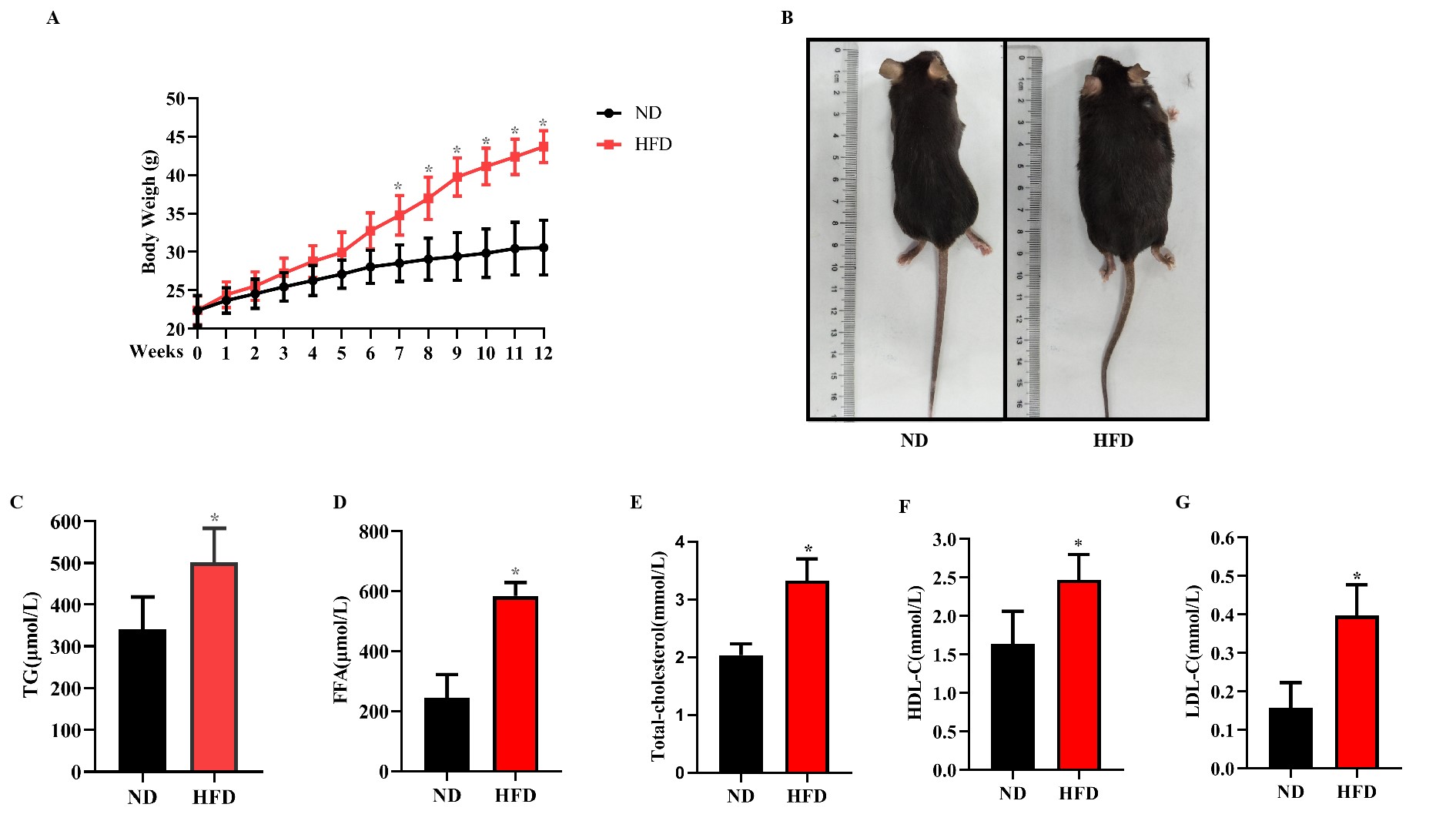
**

**Supplementary Figure. 2.** Long-chain saturated free fatty acids induced cell death in H9c2 cardiomyocytes. Cardiomyocytes were seeded onto a 24-well plate and treated with indicated doses of free fatty acids for 12 h. LDH release in the culture medium was measured and cell viability assessed using MTT assays after treatment with various fatty acids. (A-B) Cardiomyocytes were treated with 0.2, 0.4, and 0.8 mM palmitic acid (PA). (C-D) Cardiomyocytes were treated with 0.2, 0.4, and 0.8 mM stearic acid (SA). (E-F) Cardiomyocytes were treated with 0.2, 0.4, and 0.8 mM oleic acid (OA). All values are mean ± SD of 3 or more independent tests. *P* < 0.05 indicates statistical significance

.**
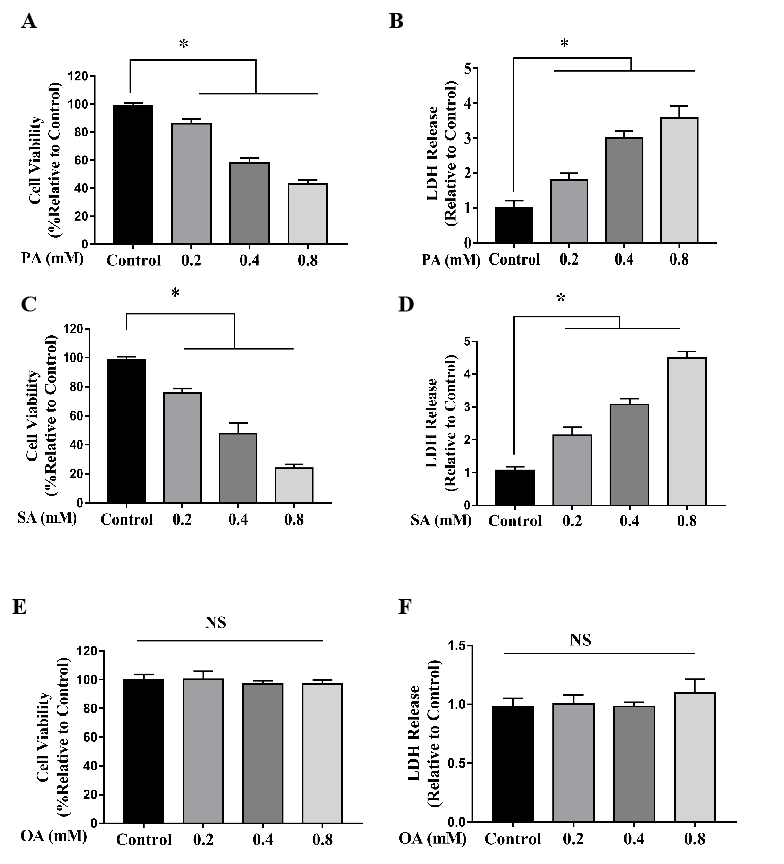
**
